# The Role of 3D Printing in the Development of a Catalytic System for the Heterogeneous Fenton Process

**DOI:** 10.3390/polym15030580

**Published:** 2023-01-22

**Authors:** Lucia D’Accolti, Alessia De Cataldo, Francesco Montagna, Carola Esposito Corcione, Alfonso Maffezzoli

**Affiliations:** 1 Department of Chemistry, University of Bari, Orabona 4, 70125 Bari, Italy; 2 Department of Engineering for Innovation, University of Salento, Arnesano, 73100 Lecce, Italy; 3 National Interuniversity Consortium of Materials Science and Technology, University of Salento, Arnesano, 73100 Lecce, Italy

**Keywords:** heterogeneous catalysis, 3D printing, green chemistry

## Abstract

Recycling of catalysts is often performed. Additive manufacturing (AM) received increasing attention in recent years in various fields such as engineering and medicine, among others. More recently, the fabrication of three-dimensional objects used as scaffolds in heterogeneous catalysis has shown innumerable advantages, such as easier handling and waste reduction, both leading to a reduction in times and costs. In this work, the fabrication and use of 3D-printed recyclable polylactic acid (PLA) scaffolds coated with an iron oxide active catalyst for Fenton reactions applied to aromatic model molecules, is presented. These molecules are representative of a wider class of intractable organic compounds, often present in industrial wastewater. The 3D-printed PLA-coated scaffolds were also tested using an industrial wastewater, determining the chemical oxygen demand (COD). The catalyst is characterized using electron microscopy coupled to elemental analysis (SEM/EDX) and thermogravimetry, demonstrating that coating leach is very limited, and it can be easily recovered and reused many times.

## 1. Introduction

One of the most important aspects of catalysis is the development of new catalysts capable of making chemical processes with low environmental impact possible. Among the most attractive methodologies, heterogeneous catalysis, in which the catalyst is immobilized on solid supports, has a prominent role. According to this method, the separation of the catalyst using filtration and/or centrifugation of the reaction mixture, its recovery, and possible reuse makes catalysts economically advantageous, with a consequent important impact on the environmental footprint of the process.

A further improvement of the processes in heterogeneous catalysis is represented by the possibility of building catalysts through the three-dimensional (3D) printing, first described in 1986 by Charles Hull [1]. More research has occurred since, further improving the environmental impact and the economic advantages of the process [2]. An important example was reported in 2016 by Sotelo and Gil, which for the first time, applied a 3D catalyst in the Ullmann reaction. [3]. Following this first paper, various approaches have been used for the construction of 3D catalysts, as monolithic structures, made of ceramic or zeolites, using polymeric materials as templates [3,4,5,6,7,8], which must be then functionalized with the catalyst. Alternatively, it could be possible to prepare the catalyst using the polymeric material as 3D scaffolds to support the catalyst [9].

The application of the additive manufacturing (AM) technologies allows the production of 3D objects by additive processes rather than subtracting/machining material from a larger bulk [10], in five different stages: (1) computer-aided design (CAD) of the structure; (2) converting the model file in surface tessellation language (STL) format file; (3) orientation and slicing operations; (4) G-code format file creation; (5) fabrication by adding the selected material layer-by-layer (also called 3D printing). CAD helps to precisely control size and geometry of objects, so it is possible to fabricate complex structures with very good accuracy [11]. Since AM technologies build the object adding material layer-by-layer [12], they do not produce the typical waste generated by traditional methods of subtractive production. For these reasons, chemical and materials engineers who are involved in the design and manufacture of artifacts have contributed to the development of this technology and of many different suitable materials. Several 3D printing techniques have been developed up to now and some of them are well-established [11,13], such as fused deposition modeling (FDM), which is, nowadays, the most used, because it is inexpensive, and many printers are available for private and professional use. In FDM, the most common materials are polymers such as acrylonitrile butadiene styrene (ABS), polylactic acid (PLA), and high-impact polystyrene (HIPS). In recent years, polylactic acid (PLA) has become one of the most used polymers because it offers many advantages: (I) it derives from renewable resources; (II) it is biodegradable and compostable; (III) it does not generate toxic fumes during the printing process [2]; (IV) it is chemically inert to water and organic solvents [14], which makes it particularly useful for applications in catalysis, from a green chemistry perspective. To produce the 3D-printed model catalysts using PLA, the most common techniques are mechanically mixed dyes, soluble drugs, and metal salt precursors mixed with the polymer before hot melting extrusion, but it is certainly much easier to use the models as scaffold for a catalytic solid coating. [15].

In the light of these considerations and continuing in our efforts in green chemistry [16,17,18], this work focused on the 3D printing of woodpile-like geometry PLA scaffolds, as a support coated by ferrous oxides suitable for the catalysis of the Fenton process. The chosen architecture adds advantages to the total process because it increases the exposed surface area of the 3D-printed object [19] and it is also easy to print [20]. This approach is highly innovative and has few precedents, as 3D-printed porous Mg/Cu catalysts used in the degradation of rhodamine B in a Fenton-like catalyst approach [21], and a more recent paper showed it is possible to prepare iron-impregnated PLA for the 3D-printed milli-fluidic reactors able to give the Fenton reaction in abatement of methylene blue as a model compound [15].

In recent years, advanced oxidation processes (AOPs) have been studied as a promising organic wastewater treatment method based on the in situ generation of hydroxyl radicals (HO), which have a strong oxidation capacity (standard potential = 2.80 V versus standard hydrogen electrode) [22]. The two reactions involved in the classical Fenton process are:

Fe^+2^ + H_2_O_2_ = Fe^+3^ + HO^●^ + OH^−^ (1)

Fe^+3^ + H_2_O_2_ = Fe^+2^ + HOO^●^ + H ^+^ (2)

HO^●^ + pollutant = CO_2_ + H_2_O (3)

H_2_O_2_ oxidizes Fe(II) to give Fe(III), a hydroxyl radical, and a hydroxyl ion in the first reaction. The Fe(III) is reduced to Fe(II) by another molecule of H_2_O_2_, giving a hydroperoxyl radical with a proton. The hydroxyl radical can give nonselective oxidation of organic compounds. [23,24]. Based on the above advantages, the Fenton process has been applied to treat many kinds of wastewater (WW) such as from olive-oil mills and laboratories, textiles, pesticide WW, cosmetics [25], dyes [26,27], fermentation brine from green olives, and pharmaceuticals. The Fenton and related reactions are viewed as potentially convenient and economical ways to generate oxidizing species for treating chemical wastes. Compared to other bulk oxidants, hydrogen peroxide is inexpensive, safe, and easy to handle, and poses no lasting environmental threat since it readily decomposes to water and oxygen. Likewise, iron is comparatively inexpensive, safe, and environmentally friendly [28]. The principal disadvantage of using this technique in the treatment of wastewater is represented by the need to remove the ferrous sludge that is generated, before its discharge [29].

To solve the problem of sludge, one possibility was the use of a packed-bed heterogeneous catalysis reactor: for example, by using the Fe(II) on carbon black it is possible to obtain a total organic carbon (TOC) removal of ca. 47%, with the reactor operated at 50 °C [30], although even in heterogeneous catalysis some disadvantages remain such as, for example, the high costs of the plant and its regeneration [31].

A suitable monolithic heterogeneous catalyst represents a strategy to avoid sludge formation. Moreover, it is also easily separable from the wastewater stream and easily recoverable and reusable for consecutive cycles of WW treatments, without losing its efficiency and efficacy, with a saving of materials and resources used in its production. In this work, the effectiveness of the 3D-printed scaffolds coated with ferrous oxide catalysts was tested in the Fenton treatment of aqueous solutions of benzothiazole (an emerging pollutant), resorcinol (an organic molecule representative of a wider class of recalcitrant compounds), and methylene blue (a dye often present in industrial wastewater). Finally, a sample of real wastewater kindly provided by an Italian steel industry factory was treated.

## 2. Materials and Methods

### 2.1. Materials

Aluminum oxide (Al_2_O_3_; molecular weight 101.96 g/mol) and sodium silicate solution (Na_2_SiO_4_) were purchased from Honeywell. Ferrous sulphate heptahydrate (FeSO_4_·7H_2_O; molecular weight 278.05 g/mol) and iron (II) chloride (FeCl_2_; molecular weight 126.75 g/mol), used as a source of Fe^2+^, were purchased from Carlo Erba and Sigma Aldrich, respectively. Isopropyl alcohol (C_3_H_8_O; molecular weight 60.096 g/mol) was purchased from J.T. Baker. Polyvinyl alcohol (average molecular weight 9000–10,000; 80% hydrolyzed) was purchased from Sigma-Aldrich (Milan, Italy). Sulphuric acid (H_2_SO_4_; molecular weight 98.07 g/mol; concentration in aqueous solution 96%) and sodium hydroxide (NaOH reagent grade, ≥98%) were purchased from J.T. Baker and Merck, respectively, and were used for pH adjustment. Hydrogen peroxide (H_2_O_2_; molecular weight 34.01 g/mol; assay 37%; concentration ≥ 35%) was purchased from Honeywell. Hydroxylamine hydrochloride (NH_2_OH·HCl; molecular weight 69.49 g/mol), used for catalyst regeneration, was purchased from Sigma Aldrich. Benzothiazole (C_7_H_5_NS; molecular weight 135.19 g/mol; assay ≥ 96%), resorcinol (molecular weight 110.11 g/mol; assay ≥ 99%), and methylene blue (C_16_H_18_ClN_3_S·xH_2_O; molecular weight 319.85 g/mol for anhydrous basis) were purchased from Sigma Aldrich and used as model molecules. Finally, a sample of wastewater was purchased from an Italian steel industry factory. UV–vis spectra were recorded using an Agilent Varian Cary 5000 (Turin, Italy) spectrophotometer. COD analyses were performed with QuickCOD Labservice instrument (Bologna, Italy) 

### 2.2. Catalyst Preparation

#### 2.2.1. 3D Printing of Polylactic Acid (PLA) Scaffolds

The commercial materials most commonly used with FDM printers are thermoplastic polymers, such as polycarbonate (PC), acrylonitrile butadiene styrene (ABS), polylactic acid (PLA), and polyethylene terephthalate glycol (PETG), thanks to their low melting or softening temperature, which allows them to be used in the FDM printing technique, using a maximum operating temperature of 300 °C. These polymers are characterized by good processability, versatility, and adaptability to FDM, allowing a variety of shapes and colors, imparting an adequate strength to the final 3D objects [32,33]. However, the choice of material to be used in the printing process depends on the object to be printed, its use, and its morphological/aesthetic characteristics. Usually, the most commonly used material for 3D printing by FDM is polylactic acid (PLA), a bio-based, biodegradable, and biocompatible thermoplastic polymer that is widely used in the food, medical, cosmetic, agro-industrial, textile, and art sectors. Derived from renewable resources such as maize, it has a production capacity of over 140,000 tons/year and it is hydrolytically degradable. Its main characteristics are: extrusion temperature ranging from 160 °C to 230 °C, tensile strength between 35 MPa and 65 MPa, flexural strength up to 97 Mpa, and Young's modulus of 2.0–2.3 GPa. However, PLA possess some disadvantages, such as its great sensitivity to high temperatures (from 200 °C), easy degradability (3–6 months depending on the environmental conditions, size and filling of the object), and hygroscopicity [32]. 

A polylactic acid (PLA) filament purchased from the company Fabbrix^®^ (Ruvo di Puglia, Bari, Italy) was used to print the catalysts. According to the technical data sheet, it has a diameter of 1.75 ± 0.05 mm, a density of 1.25 g/cm^3^, and a melt flow index (MFI) of 7–9 g/10 min at a temperature of 190 °C. PLA filament was selected for its biodegradability, low cost, and easy processability. All these characteristics could, in fact, be considered suitable for the development of heterogeneous catalysts characterized by a low environmental footprint.

The 3D printing of polylactic acid (PLA) scaffolds was carried out at the Department of Engineering for Innovation of the University of Salento, and it consisted of five steps:

1. CAD design of the structure;
2. Exporting the file in *.stl format;
3. Orientation and slicing operations;
4. Creation of the G-code file;
5. 3D printing.

**Figure 1:**
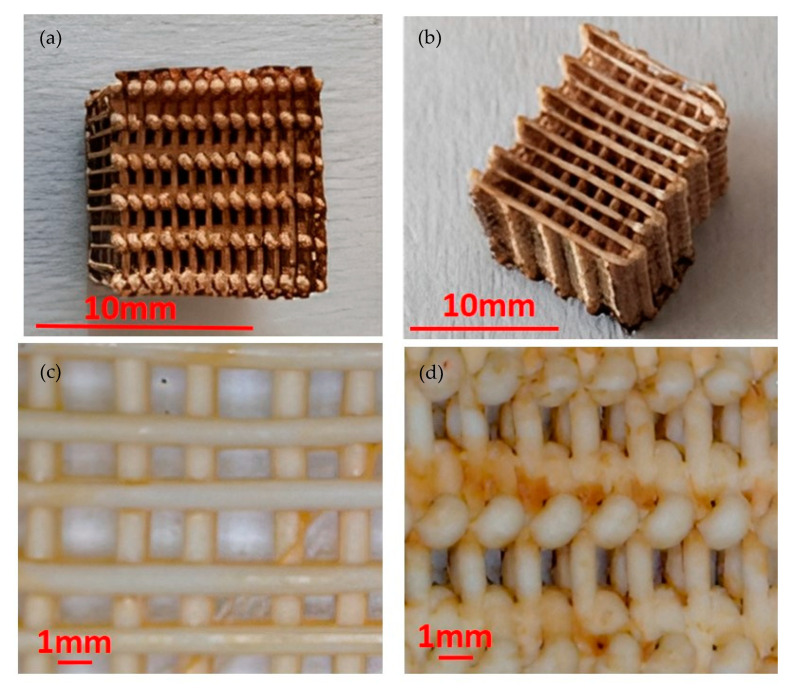
(**a**): Top view of the catalyst; (**b**) side view of the catalyst; (**c**) detail of a midplane section parallel to the building direction in 3D printer; (**d**) detail of a side view.

Fusion360 software was used for the first and second phase; for the third and fourth steps, Ultimaker Cura software was adopted. The printing of the 3D structures was performed through fused deposition modeling (FDM) using the printer Creality CP-01 and a PLA filament, white in color and with a diameter of 1.75 mm. A woodpile-shaped scaffold (Figure 1) with an external shape of a cube was printed setting the following operating conditions: nozzle diameter equal to 0.8 mm, extrusion temperature equal to 200 °C, printing speed of 60 mm/s, infill density of 30%, infill line distance of 1.1 mm, layer height of 0.4 mm. It is important to note that the selection of an adequate building strategy is very relevant to obtain the required macroporosity of the catalyst. In particular, the selection of the right infill density parameter is crucial to obtain the geometry of the samples. The obtained scaffolds, as the one shown in Figure 1a, are characterized by a weight of 0.56 + 0.02 g, a volume of 1.248 cm^3^, and a density of 0.45 g/cm^3^. The surface area, relevant for the catalytic behavior of the scaffold, is 38.30 cm^2^.

#### 2.2.2. Preparation of the Catalytic System

The printed 3D PLA scaffold was first used as a template to prepare the ceramic catalyst following a procedure reported in the literature [5]: the scaffold was first coated with a paste consisting of Al_2_O_3_ powder dispersed in sodium silicate solution and dried for 24 h at room temperature, then for another 24 h at 60 °C; finally, it was heated in a muffle up to 850 °C and then kept at 850 °C for 8 h to calcinate. The prepared monolith was a negative replica of the PLA scaffold, even if the shape and size of this sample were not replicated precisely. This monolith was then impregnated with an aqueous solution of FeSO_4_ 0. 2 M for 24 h and rapidly dried. The same procedure was then used removing the calcination phase in the muffle following the covering of the scaffold, to give the catalyst higher mechanical resistance due to the presence of the PLA skeleton. Finally, the 3D PLA template was used directly for impregnation of Fe (II) using the procedure described below:

● PHASE 1: Chemical attack of the PLA scaffold by immersion in a 1 M aqueous solution of sodium hydroxide (NaOH), under magnetic stirring on a plate heated to 65 ° C for 45 min in a sand bath;
● PHASE 2: Preparation of the following solutions:
o Aqueous solution of polyvinyl alcohol (PVA) at 2% by weight;
o Solution of FeCl_2_ in isopropanol (C_3_H_8_O) at 0.5% (3.96 × 10^−5^ M);
● PHASE 3:
o Adding 25 mL of aqueous PVA solution to 25 mL of FeCl_2_ solution in isopropanol and magnetic stirring for 2 h;
o Immersion of the catalyst in solution under magnetic stirring for 24 h;
o Dripping and transferring to an oven at 60 °C for a week.

A similar procedure was, first, successfully used by Anita Lett J et al. [34] for the fabrication and testing of a novel composite, magnesium (Mg)-doped hydroxyapatite (HAp) glazed onto polylactic acid (PLA) scaffolds, where polyvinyl alcohol (PVA) was used as a binder. Starting from this interesting result, the authors decided to modify the literature process and adapt it to the aim of this paper. PVA was selected as a binder between PLA and FeCl_2_ and its compatibility with PLA was increased by using a sodium hydroxide surface treatment. However, a solvent able to increase the compatibility between PVA and FeCl_2_ was necessary. To this aim, after testing several solvents, isopropanol was selected as the most suitable solvent for PVA and FeCl_2_. Instead, water, a more eco-compatible and economic solvent, was found for PVA.

#### 2.2.3. Characterization Methods

An AXIO-LINKAM optical microscope (Zeiss, Milano, Italy) was used for the morphological characterization of the 3D-printed catalyst. The size of the pores was obtained as an average of 10 measurements.

Scanning electron microscope (SEM) images were obtained by a Phenom XL G2 Desktop SEM instrument in high vacuum and high-resolution mode, equipped with a Gemini column and an integrated high efficiency in-lens detector. The applied acceleration voltage was 5 kV. The instrument was equipped with an EDX detector to obtain the elemental analysis of selected sample areas. The analyses were performed at least five times to verify the accuracy of the results.

The thermal stability of the samples before and after the Fenton process was determined by thermogravimetric analysis (TGA) by a TA Instrument SDT Q600 (TA Instrument, New Castle, DE, USA). About 10 mg of each sample was heated from 25 °C to 700 °C at 10 °C/min in air atmosphere. Each measurement was repeated three times.

### 2.3. Fenton Process

#### 2.3.1. Preparation of Model Molecules Solutions

Benzothiazole, resorcinol, and methylene blue were used as model molecules in bi-distilled water. A methylene blue (MB) aqueous solution (0.001 M), a benzothiazole (BTH) aqueous solution (0.74 × 10^−6^ M), and a resorcinol aqueous solution (0.01 M) were prepared.

#### 2.3.2. Batch Experiments

The selected reaction conditions were obtained by comparison with the literature on homogeneous catalysis [35]. A known volume (50 mL) of aqueous solution of molecule to be degraded was transferred to a beaker and brought to pH 3. pH adjustments were performed using sulfuric acid (96%) and sodium hydroxide (5 M). The catalyst was immersed in the bulk of the solution, stirred on a plate and, at the same time, a known volume (1 mL) of hydrogen peroxide (15–20 × 10^−3^ M, 50% wt in H_2_O, d= 1.2 g/mL) was added. The UV–vis spectra of the aqueous solutions of methylene blue at pH 3 were acquired in the range 251–800 nm, while the spectra of aqueous solutions of benzothiazole and resorcinol at pH 3 were acquired in the range of 200–500 nm. The degradation of the molecules was monitored at the wavelength at which the maximum of absorption. The catalyst was recycled using an aqueous solution of hydroxylamine hydrochloride (7, 8 × 10^−2^ M) and recycling was carried out by transferring the catalyst into a beaker and adding the hydroxylamine solution, stirring with a magnetic stirrer for about 30 min [36].

#### 2.3.3. Fenton Treatment of a Sample Wastewater

The Fenton process was used to reduce the chemical oxygen demand (COD) of a portion (50 mL) of the wastewater sample supplied by an Italian steel industry factory.

#### 2.3.4. Determination of the Chemical Oxygen Demand (COD)

The determination of the COD of the wastewater sample was carried out by the method QuickCOD. The sample to be analyzed was injected into a furnace at 1200 °C where, through a pumping system, air and nitrogen, which acts as carrier gases, are used. In the furnace the thermal reduction of oxygen in the air occurs together with the oxidation of organic and inorganic molecules present in the sample. The gaseous oxidation products are subsequently transferred to the condenser, through the action of the carrier gas. A quartz wool filter and an acid trap are placed downstream of the condenser to ensure constant conditions for the oxygen sensors. An additional filter of air allows the purification of the air, and the resulting oxygen can be measured by the sensors. Note the initial amount of oxygen added to the system (defined by the operator), as the oxygen consumed can be calculated by subtracting the amount of oxygen detected at the end of the process from that initially defined. The oxygen consumed during the reaction indirectly provides a measure of organic and inorganic pollutants present in the sample.

## 3. Results and Discussion

### 3.1. Preparation of the Catalyst

A 3D-printed structure coated by the active catalytic ferrous oxide was initially made according to the method described by Michorczyk et al. [5] for a ceramic structure. When the catalyst obtained by this method is tested in the Fenton process, it shows poor strength and crumbled in the reaction beaker, being non-reusable. The catalyst prepared following a second coating method shows an extensive occlusion of the pores generated by the 3D printing process, partially losing the shape of the 3D-printed scaffold. Furthermore, once the coated scaffold is used in the Fenton process, coating debonding occurs.

Considering these results, we decided to develop a new method that would preserve both the 3D scaffold integrity and its macroporous structure. This approach allowed repeated use of the catalyst with a negligible leaching of iron ions.

### 3.2. Catalysts Characterization

The pictures taken by optical microscope, shown in Figure 1a,b, show that the external surfaces of the coated scaffold are characterized by a brown color due to the ferrous oxide. However, the midplane of the catalyst, shown in Figure 1c, is characterized by a lower amount of ferrous, indicating that the coating treatment is more effective on the external surfaces of the scaffold. A longer treatment time should lead to a more uniform distribution of the coating. A non-fully homogeneous distribution of Fe on the external surface is shown in Figure 1d.

**Figure 2:**
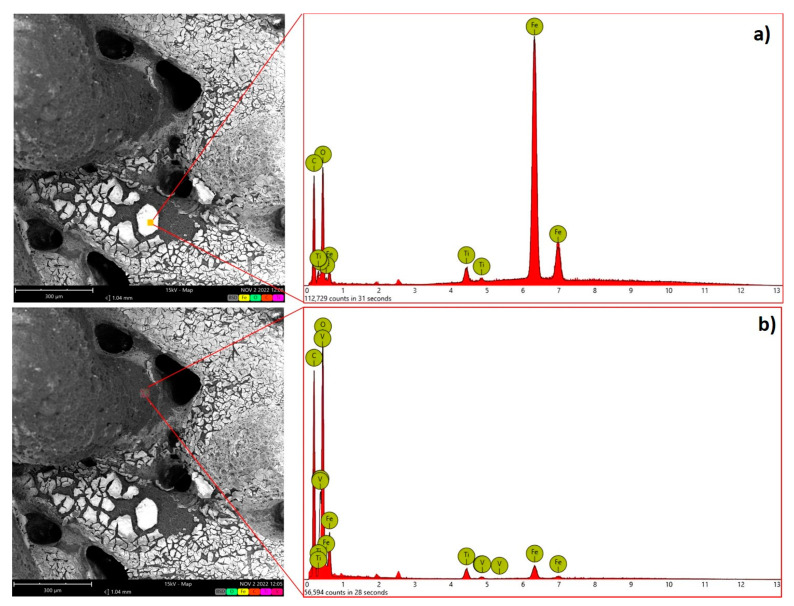
Left: SEM picture of the catalyst. Right: EDX analysis is shown at two different areas characterized by a different content of Fe. (**a**) analysis of first mapping; (**b**) analysis of second mapping.

SEM analyses carried out on the treated 3D-printed samples (Figure 2), confirm that the Fe oxide coating is not deposited homogenously. EDX analysis indicates that areas richer in Fe are present at the catalyst surface, as evidenced by the average atomic concentration reported in Table 1.

### 3.3. Fenton Treatment of Model Molecules Solutions

The extensive application of dyes, aromatic compounds (often derived from phenol), and BTHs in industrial and consumer products has led to widespread contamination of the environment. Different methods for these pollutants’ degradation have been reported [37,38], but, in most cases, oxidative processes based on H_2_O_2_ and UV are used, which are not practicable at an industrial level [38]. To verify the potential of 3D-printed catalysts in Fenton processes, preliminarily methylene blue, which can be considered as a model compound for many degradation studies [35,38], was used. Then benzothiazole, as a model molecule for secondary pollutants (antibiotics, drugs, etc.) and resorcinol, as a model for phenols pollutants, were also tested.

**Figure 3:**
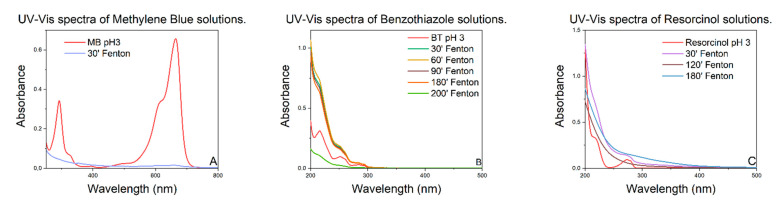
UV–vis spectra of model molecules solutions, over time.

Figure 3 shows the UV–vis spectra of the experiments carried out on methylene blue (Figure 3A), benzothiazole (Figure 3B), and resorcinol (Figure 3C). It is evident that the chosen catalyst can completely degrade the chosen model molecules in a time between 30 and 200 min.

In particular, the UV–vis spectra of the aqueous solutions of methylene blue at pH 3 (Figure 3A) show the complete degradation of the dye within only 30 min after the addition of the hydrogen peroxide. For benzothiazole and resorcinol, which are more intractable than methylene blue, the complete degradation requires 200 min and 120 min, respectively.

As reported below in Table 2, these results compared with the literature show that the 3D-printed PLA catalyst presents a catalytic activity comparable to that of more complex systems. For example, the degradation of methylene blue (row# 1) occurs with the same efficiency of more complex heterogeneous catalysts, such as, for example, an Fe- based amorphous alloy [39]. Similar data could be obtain using the Fe–PLA impregnate (row# 2); in this case, the complete degradation occurs in 20 min [16].

On the other hand, when benzothiazole degradation (row# 4) is conducted in a homogeneous condition, only 20 percent is degraded in the first hour [37]. Our result is comparable with the photo-Fenton processes (row# 5) conducted in the presence of β-cyclodextrin, useful to improve the solubility, in which the reaction proceeds up to 80% in 60 min [37]. 

Finally, the resorcinol degrades almost completely in 120 min, similarly to what happens in photo-Fenton processes (row# 7) [40].

**Figure 4:**
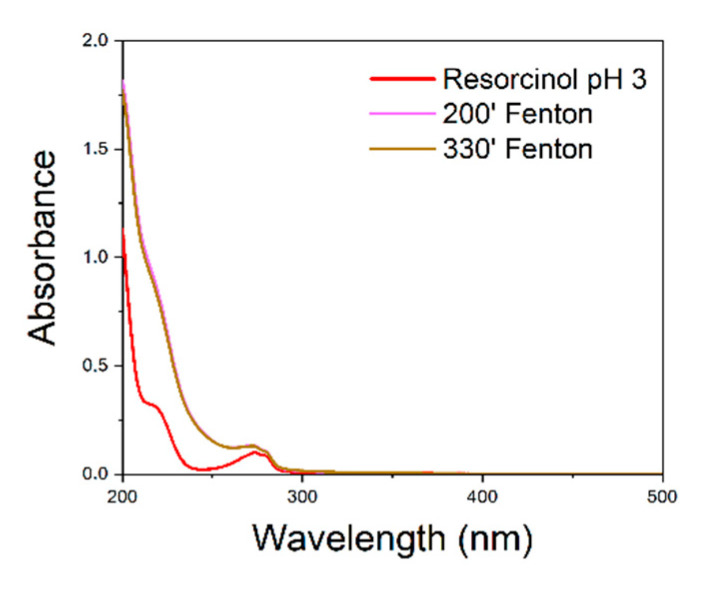
UV–vis spectra of Fenton-processed resorcinol solution using the unregenerated catalyst for the second time. UV–vis spectra recorded after 200 and 330 min overlap.

To verify the recyclability of the catalyst, resorcinol reaction was chosen because of the lower degradation time compared to benzothiazole. The reaction was carried out for a second time using the catalyst as it was, without regeneration. The results are shown in figure (Figure 4):

It is evident that the degradation of resorcinol does not take place using the catalyst, without any regeneration treatment.

**Figure 5:**
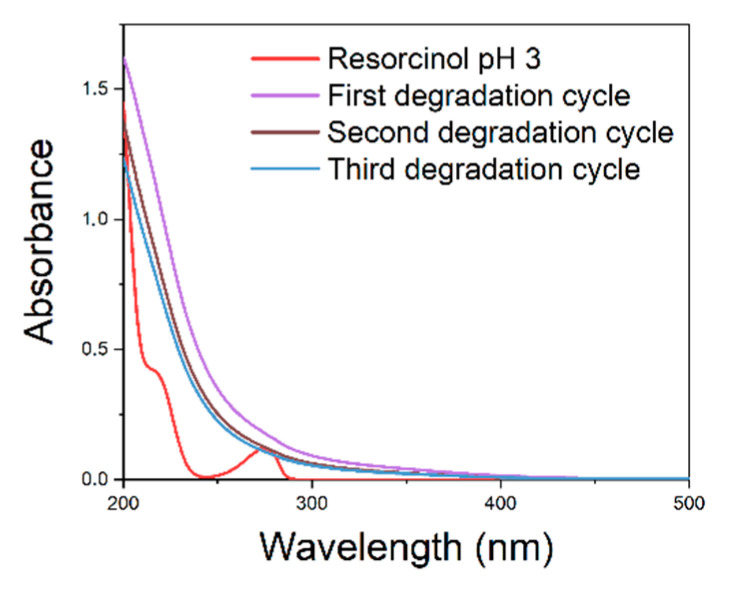
UV–vis spectra of Fenton-processed resorcinol solution using the catalyst after regeneration with hydroxylamine hydrochloride.

To overcome this drawback, the catalyst was regenerated using hydroxylamine hydrochloride, as reported in the literature [36]. In particular, the catalyst was immersed in a hydroxylamine hydrochloride aqueous solution (7.8 × 10^−2^ M) for about 30 min, and briefly dried, then being ready to be used. The catalyst after regeneration was tested three times, as shown in Figure 5:

Figure 5 shows that the resorcinol degradation takes place in three consecutive experiments carried out using the same catalyst after regeneration with hydroxylamine hydrochloride. Therefore, we can conclude that during the Fenton reaction there is no relevant Fe(II/III) leaching affecting the catalytic activity in three consecutive cycles of reactions and regenerations.

### 3.4. Fenton Treatment of a Sample Wastewater

The catalyst was finally used in the Fenton process for the abatement of organic and inorganic molecules present in a sample of wastewater made available by an Italian steel industry. To verify the effectiveness of the catalyst, an analysis of the Chemical Oxygen Demand (COD) of the sample was carried out before treatment and after 30 min of treatment, using the Quick COD method, that showed the amount of pollutant at retention time 1019 min 34% disappeared after 30 min.

**Figure 6:**
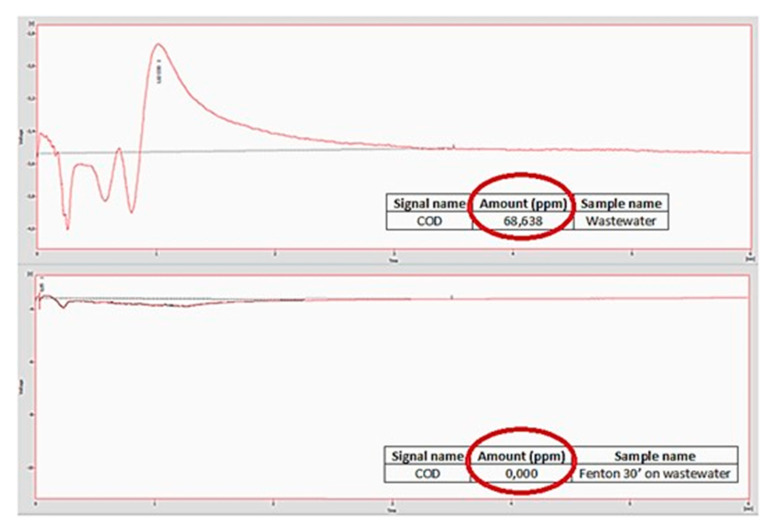
Comparison between COD of wastewater (**top**) and COD of wastewater after treatment with Fenton reagent after 30 min (**bottom**).

As shown in Figure 6, comparing the two plots and the ppm of residual organic compounds circled with red, a complete abatement of the species present in the sample is achieved within just 30 min after adding the reagents and the catalyst. We can, in fact, observe that the total amount of residual organic compounds in the sample of wastewater (in the plot above) is equal to 68,638 ppm, while the amount of the residual organic compounds after only 30 min of Fenton treatment on the same sample is equal to 0 ppm.

**Figure 7:**
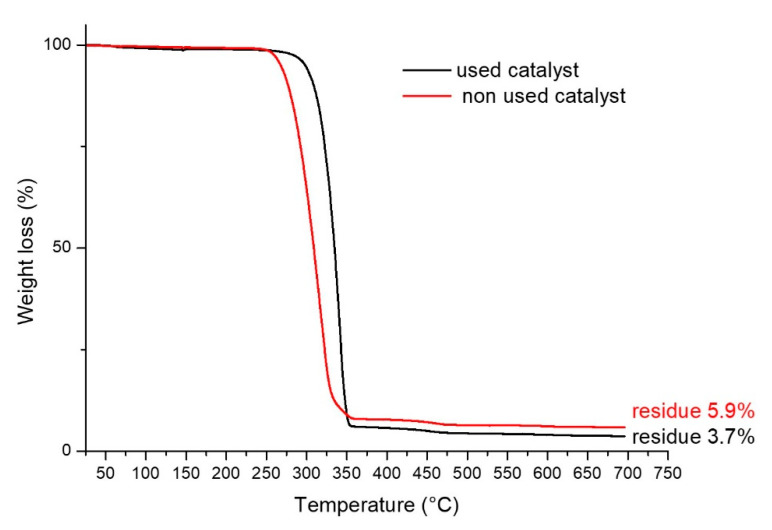
Thermogravimetric analysis of the catalyst before its use and after 3 reaction and regeneration cycles.

The TGA curves reported in Figure 7 and the respective weight loss results reported in the same figure, evidence a small difference between the solid residue of the catalyst before its use and after three reaction and regeneration cycles, indicating a limited leaching of Fe. This result supports the effectiveness of the catalyst in multiple cycles, as reported above.

## 4. Conclusions

In summary, a 3D-printed PLA catalyst coated by ferrous oxide was capable to oxidize several organic compounds. The proposed heterogenous catalytic system shows several advantages, the key ones being summarized as follows:

- Simple and reliable preparation of a monolithic catalyst characterized by a complex geometry by 3D printing followed by a simple coating procedure;
- High efficiency and selectivity of the oxidations provide highly degradation yield and avoiding costly and time-consuming work-up procedures;
- The catalyst can be recovered and reused after regeneration, thus, increasing the effectiveness of the process;
- Our method resolves the problem of the sludge normally obtained using the traditional homogeneous and heterogeneous reaction, reducing the wastes according of the rules of green chemistry;
- Furthermore, the study conducted on industrial wastewaters shows how it can also be applied in complex mixtures, which represents an advance compared to current studies on catalyzed oxidation processes.

In conclusion, the use of 3D-printed catalysts opens the way to new applications, especially in the environmental field, as shown comparing the presented results to those reported in the literature.

## Figures and Tables

**Table 1:** Average atomic concentration of the main elements as determined by EDX analysis reported in Figure 2a,b.

Element Symbol	Atomic Concentration EDX (Figure 2a)	Atomic Concentration EDX (Figure 2b)
O	80 ± 2	60 ± 1
Fe	4 ± 0.5	30 ± 0.8
C	12 ± 1	2 ± 0.5
Element Symbol	Weight Conc.	Oxide Symbol
Fe	83.49	
O	12.11	
C	2.35	
Ti	2.05	

**Table 2:** Comparison of the efficiency of the 3D PLA catalyst with different results in the literature.

Row #	Catalyst	Substrate	Time (min)	Temperature (K)	Conversion (%)	Ref #
1	Fe_63_Cr_5_Nb^4^Y_6_B_22_	MB	20	70	100	39
2	Fe(II)–PLA impregnated stirred caps	MB	20	25	100	15
3	Fe(II)–3D-printed PLA	MB	30	25	100	Our work
4	Fenton	BTH	60	25	20	37
5	Photo-Fenton	BTH	60	25	80	37
6	Fe(II)–3D-printed PLA	BTH	200	25	100	Our work
7	Photo-Fenton	Resorcinol	120–240	25	100	40
8	Fe(II)–3D-printed PLA	Resorcinol	120	25	100	Our work

## Data Availability

No new data were created or analyzed in this study. Data sharing is not applicable to this article.

## References

[1] Hull C.W. (1986). Apparatus for Production of Three-Dimensional Objects by Stereolithography. U.S. Patent.

[2] Benaglia M., Rossi S., Puglisi A. (2018). Additive manufacturing technologies: 3D printing in organic synthesis. ChemCatChem.

[3] Tubío C.R., Azuaje J., Escalante L., Coelho A., Guitián F., Sotelo E., Gil A. (2016). 3D printing of a heterogeneous copper-based catalyst. J. Catal..

[4] Bogdan E., Michorczyk P. (2020). 3D printing in heterogeneous catalysis—The state of the art. Materials.

[5] Michorczyk P., Hedrzak E., Wegrzyniak A. (2016). Preparation of monolitich catalysts using 3D Printed templates for oxidative coupling of methane. J. Mater. Chem. A.

[6] Stuecker J.N., Miller J.E., Ferrizz R.E., Mudd J.E., Cesarano J. (2004). Advanced support structures for enhanced catalytic activity. Ind. Eng. Chem. Res..

[7] Ferrizz R.M., Stuecker J.N., Cesarano J., Miller J.E. (2005). Monolitich supports with unique geometries and enhanced mass transfer. Ind. Eng. Chem. Res..

[8] Li X., Rezaei F., Rownaghi A.A. (2018). 3D-printed zeolite monoliths with hierarchical porosity for selective methanol to light olefin reaction. React. Chem. Eng..

[9] Chaparro-Garnica C.Y., Davó-Quiñonero A., Bailón-García E., Lozano-Castelló D., Bueno-López A. (2019). Design of Monolithic Supports by 3D Printing for Its Application in the Preferential Oxidation of CO (CO-PrOx). ACS Appl. Mater. Interfaces.

[10] Hurt C., Brandt M., Priya S.S., Bhatelia T., Patel J., Selvakannan P.R., Bhargava S. (2017). Combining additive manufacturing and catalysis: A review. Catal. Sci. Technol..

[11] Wang X., Jiang M., Zhou Z., Gou J., Hui D. (2017). 3D printing of polymer matrix composites: A review and prospective. Compos. B Eng..

[12] (2012). Standard Terminology for Additive Manufacturing Technologies.

[13] Zhou X., Liu C. (2017). Three-dimensional printing for catalytic applications: Current status and perspectives. Adv. Funct. Mater..

[14] Gordeev E.G., Degtyareva E.S., Ananikov V.P. (2016). Analysis of 3D printing possibilities for the development of practical applications in synthetic organic chemistry. Russ. Chem. Bull..

[15] Flores D., Noboa J., Tarapues M., Vizuete K., Debut A., Bejarano L., Streitwieser D.A., Ponce S. (2022). Simple Preparation of Metal-Impregnated FDM 3D-Printed Structures. Micromachines.

[16] Tarzia A., Montanaro J., Casiello M., Annese C., Nacci A., Maffezzoli A. (2018). Synthesis, curing and properties of an epoxy resin derived from gallic acid. Bioresources.

[17] Pantone V., Annese C., Fusco C., Fini P., Nacci A., Russo A., D’Accolti L. (2017). One-Pot Conversion of Epoxidized Soybean Oil (ESO) into Soy-Based Polyurethanes by MoCl2O2 Catalysis. Molecules.

[18] Casiello M., Catucci L., Fracassi F., Fusco C., Laurenza A.G., Di Bitonto L., Pastore C., D’Accolti L., Nacci A. (2019). ZnO/Ionic Liquid Catalyzed Biodiesel Production from Renewable and Waste Lipids as Feedstocks. Catalyst.

[19] Hartings M.R., Ahmed Z. (2019). Chemistry from 3D printed Objects. Nat. Rev. Chem..

[20] Maldovan M., Thomas E.L., Carter C.W. (2004). Layer-by-layer diamond-like woodpile structure with a large photonic band gap. Appl. Phys. Lett..

[21] Yang C., Zhang C., Chen Z.-J., Li Y., Yan W.-Y., Yu H.-B., Liu L. (2021). Three-dimensional hierarchical porous structures of metallic glass/copper composite catalysts by 3D printing for efficient wastewater treatments. ACS Appl. Mater. Interfaces.

[22] Watwe V.S., Kulkarni S.D., Preeti S.K. (2021). Cr(VI)-mediated homogeneous Fenton oxidation for decolorization of methylene blue dye: Sludge free and pertinent to a wide pH range. ACS Omega.

[23] Neyens E., Baeyens J. (2003). A review of classic Fenton’s peroxidation as an advanced oxidation technique. J. Hazard. Mater. B.

[24] Sun S.P., Zeng X., Li C., Lemley A.T. (2014). Enhanced heterogeneous and homogeneous Fenton-like degradation of carbamazepine by nano-Fe3O4/H2O2 with nitrilotriacetic acid. Chem. Eng. J..

[25] Bautista P., Mohedano A.F., Gilarranz M.A., Casas J.A., Rodriguez J.J. (2007). Application of Fenton oxidation to cosmetic wastewater treatment. J. Hazard. Mater..

[26] Kušic H., Bozic A.L., Koprivanac N. (2007). Fenton type processes for minimization of organic content in coloured wastewaters: Part I: Processes optimization. Dyes Pigm..

[27] Ramirez J.H., Costa C.A., Madeira L.M. (2005). Experimental design to optimize the degradation of the synthetic dye Orange II using Fenton’s reagent. Catal. Today.

[28] Pignatello J.J., Oliveros E., MacKay A. (2006). Advanced oxidation processes for organic contaminant destruction based on the Fenton reaction and related chemistry. Crit. Rev. Environ. Sci. Technol..

[29] Kavitha V., Palanivelu K. (2004). The role of ferrous ion in Fenton and photo-Fenton processes for the degradation of phenol. Chemosphere.

[30] Mesquita I., Matos L.C., Duarte F., Maldonado-Hòdar F.J., Mendes A., Madeira L.M. (2012). Treatment of azo dye-containing wastewater by a Fenton-like process in a continuous packed-bed reactor filled with activated carbon. J. Hazard. Mater..

[31] Argyle M.D., Bartholomew C.H. (2015). Heterogeneous Catalyst Deactivation and Regeneration: A Review. Catalysts.

[32] Fico D., Rizzo D., Casciaro R., Esposito Corcione C. (2022). A Review of Polymer-Based Materials for Fused Filament Fabrication (FFF): Focus on Sustainability and Recycled Materials. Polymers.

[33] Algarni M., Ghazali S. (2021). Comparative Study of the Sensitivity of Pla, Abs, Peek, and Petg’s Mechanical Properties to Fdm Printing Process Parameters. Crystals.

[34] Anita Lett J., Sagadevan S., Léonard E., Fatimah I., Motalib Hossain M.A., Mohammad F., Al-Lohedan H.A., Paiman S., Alshahateet S.F., Abd Razak S.I. (2021). Bone tissue engineering potentials of 3D printed magnesium-hydroxyapatite in polylactic acid composite scaffolds. Artif. Organs.

[35] Wanga N., Zheng T., Zhang G., Wanga P. (2016). A review on Fenton-like processes for organic wastewater treatment. J. Environ. Chem. Eng..

[36] Zheng Y., Xie W., Yuan S. (2022). Hydroxylamine Promoted Fe(III) Reduction in H2O2/Soil Sys-tems for Phenol Degradation. Environ. Sci. Pollut. Res..

[37] Reynoso A., Zizzias J., Sacchetto J., Gambetta C., Natera J., Massad W.A. (2021). Complete benzothiazole elimination by the solar photo-Fenton process in aqueous and β-cyclodextrin solutions. New. J. Chem..

[38] Antonopoulou M., Evgenidou E., Lambropoulou D., Konstantinou I. (2014). A review on advanced oxidation processes for the removal of taste and odor compounds from aqueous media. Water. Res..

[39] Yang W., Wang Q., Li W., Xue L., Liu H., Zhou J., Mo J., Shen B. (2019). A novel thermal-tuning Fe-based amorphous alloy for automatically recycled methylene blue degradation. Mater. Des..

[40] Barona J.F., Morales D.F., Gonzàlez-Bahamòn L.F., Pulgarìn C., Benìtez L.N. (2015). Shift from heterogeneous to homogeneous catalysis during resorcinol degradation using the solar photo-Fenton process initiated at circumneutral pH. Appl. Catal. B Environ..

